# On the feasibility of a quantum sensing protocol designed with electrically controlled spins in silicon quantum dots

**DOI:** 10.1039/d5ra01109d

**Published:** 2025-04-17

**Authors:** Hoon Ryu, Kum Won Cho, Junghee Ryu

**Affiliations:** a School of Computer Engineering, Kumoh National Institute of Technology Gumi Gyeongsangbuk-do 39177 Republic of Korea elec1020@kumoh.ac.kr +82-54-478-7534; b Supercomputing Center, Kumoh National Institute of Technology Gumi Gyeongsangbuk-do 39177 Republic of Korea kwcho@kumoh.ac.kr; c Center for Quantum Information R&D, Korea Institute of Science and Technology Information Daejeon 34141 Republic of Korea; d Division of Quantum Information, University of Science and Technology Daejeon 34113 Republic of Korea

## Abstract

Though electron spins in electrically defined silicon (Si) quantum dot systems have been extensively employed for physical realization of quantum processing units, their application to quantum sensing has not been active compared to the case of photonic qubits and nitrogen-vacancy spins in diamonds. This work presents a comprehensive study on the feasibility of Si quantum dot structures as a physical platform for implementation of a sensing protocol for magnetic fields. To examine sensing operations at a systematic level, we adopt in-house device simulations taking a Si double quantum dot (DQD) system as a target device where the confinement of electron spins is controlled with electrical biases in a Si/Si-germanium heterostructure. Simulation results demonstrate the fairly nice utility of the Si DQD platform for detecting externally presented static magnetic fields, and, more notably, reveal that sensing operations are not quite vulnerable to charge noise that is omnipresent in solid materials. As a rare study that presents in-depth discussion on operations of quantum sensing units at a device-level based on computational modeling, this work can deliver practical insights for potential designs of sensing units with electron spins in Si devices.

## Abstract 2

A type-II quantum sensing unit for magnetic field is studied at a device level with electrically defined quantum dots in silicon. A systematic analysis on control engineering and performance is presented to accelerate potential device designs.

## Graphical abstract

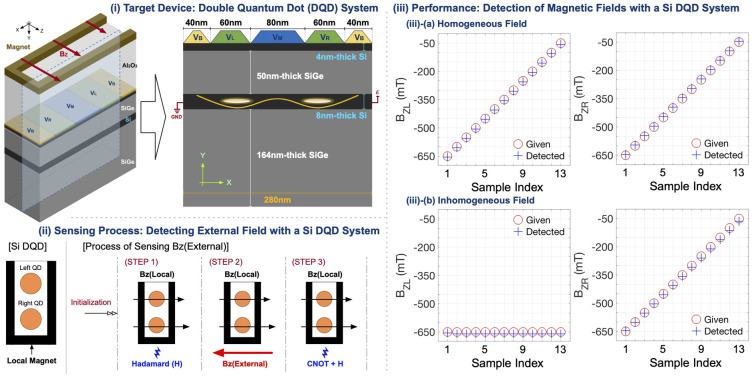

## 1 Introduction

Electron spins in silicon (Si) have been regarded as a strong physical mechanism for encoding quantum bits (qubits) since their coherence time is known to be able to reach up to several hundred milliseconds in well purified Si^28^ wafers,^1–3^ and due to the potential advantage that classical control hardware can be easily integrated with industrial-standard lithographical processes. Since the concept of controllable logic operations with electron spins in electrically controlled quantum dot (QD) structures was proposed by Loss and DiVincenzo,^4^ experimentalists have put significant efforts to realize logical building blocks for quantum computing in a Si QD system, such that today's single-qubit rotations can be conducted in ∼100 nanoseconds (ns) with a fidelity larger than 99.9%.^2,5,6^ Fast 2-qubit gating blocks are also available so SWAP, controlled-Z (CZ), and controlled-X (CNOT) logic have been demonstrated with a fidelity larger than 98%.^7–10^ Fully programmable processors have been also reported with Si QD systems,^11–13^ where the most up-do-date technical progress involves a 6-qubit quantum processing unit (QPU)^13^ that is a little behind in terms of qubit sizes compared to the world-leading status achieved with transmon qubits in superconductors^14,15^ and trapped-ion qubits.^16,17^

As a different class of applications from computations, qubits can be used for sensing operations, *i.e.* to detect or measure unknown physical quantities. According to a seminal review article written by Degen *et al.*,^18^ the most broad definition of quantum sensors includes devices that use quantum objects (*e.g.* characterized by quantized energy levels) to measure physical quantities (Type-I), so this may not necessarily limit the scope of quantum sensors to the cases that use qubits (III–V quantum dot photodetectors^19,20^ belong to the Type-I sensors though they do not control qubits to detect light of a certain characteristic wavelength). In a narrower sense, the Type-II class of quantum sensors indicate devices that use quantum coherence of qubits. With no doubt, the most relevant example of Type-II quantum sensors should be nitrogen-vacancy centers in diamonds,^21,22^ which employ temporally created superposition states for coherent sensing of magnetic and electric fields.^23–25^ The most strict definition of quantum sensors applies to the Type-III class. Having the potential to outperform classical sensors pushing the measurement accuracy to the Heisenberg limit,^26,27^ Type-III devices use entangled qubits as a sensing source and has been mainly carried with photonic and cold-atom systems.^26,28–31^ In contrast to its extensive employment for quantum computations, however, the Si QD platform have been rarely employed for sensing devices though electron spins in well purified Si^28^ are suitable to implement sensing states due to their long coherence time.^1,32^ In a broad perspective of the sensing application, QDs in Si so far have been mainly studied to design spin-readout protocols,^33,34^ whose focal functionality is to detect the spin-induced variation in currents using electron states confined to phosphorus dopants. So, sensing units based on manipulation of quantum information created with electron spins in Si QDs still remain in a conceptual level, motivating studies for systematic operations to ascend to a device application level.

Accordingly, here we investigate the feasibility of the Si QD system to implement a simple Type-II sensing protocol for magnetic fields. The sensing operation of a realistically sized Si double QD (DQD) system based on a Si/Si-germanium (SiGe) heterostructure is rigorously studied with device simulations that use electronic calculations with a parabolic effective mass model and bulk physics in a multi-scale manner.^35–37^ Through a self-consistent determination of bias-dependent electric fields and corresponding time-responses of electron spins, we secure a set of control signals with which a sensing source can be prepared and evolve to the final state containing the information of target quantities to be detected, and computationally confirm that sensing operations generally produce fairly nice results even when the target device suffers from non-negligible charge noise that exists in any solid structures and is in principle hard to be suppressed.^38–40^ Though this work is purely computational, the presented modeling results are solid enough to demonstrate that electrically defined QDs in Si can serve as a sound physical platform to develop quantum sensors, delivering practical guidelines for potential device designs.

## 2 Methods

### 2.1 Target system and device simulation

**Fig. 1:**
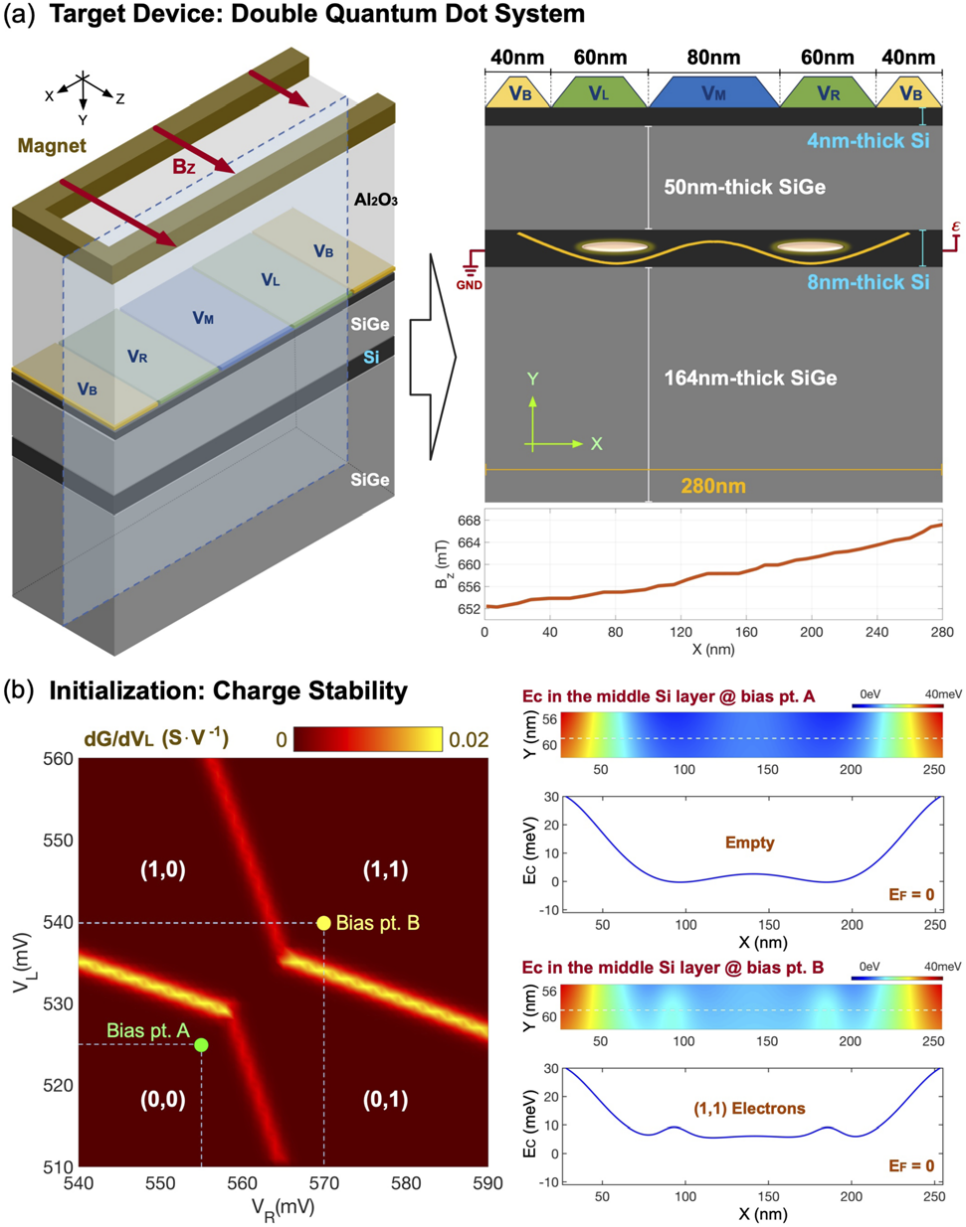
(a) The Si DQD structure that is used as a simulation target. The system controls confinement of electron spins in the middle (8 nm-thick) Si layer with DC biases imposed on top electrodes. Since the device is normally quite long along the *Z*-direction, we adopt a 2D simulation domain assuming the structure is periodic along that direction. The laterally inhomogeneous magnetic field, which is generated from a micromagnet in reality, is incorporated into the device Hamiltonian. (b) The charge stability diagram modeled at (*V*_B_, *V*_M_) = (200 mV, 400 mV). As we increase *V*_L_ and *V*_R_, spin states in QDs continue to shift lower in energy and, eventually, the ground state of each QD becomes occupied with an electron. Once the ground state is filled, the potential valley disappears due to electron screening. At (*V*_L_, *V*_R_) = (540 mV, 570 mV) (Bias pt. B), both QDs are filled with an electron and the device is initialized to a 2-qubit |↓↓〉 state satisfying the symmetric biasing condition.

Fig. 1(a) shows the Si DQD device that we target to study in this work. Taking the system reported by Zajac *et al.*^41^ as a reference, we model a Si/Si_0.7_Ge_0.3_ heterostructure where the confined electron spins reside in the 8 nm-thick middle Si layer. Here, the vertical (along the *Y*-direction) confinement is naturally created with band-offsets among Si and SiGe layers but the lateral (along the *X*-direction) one is electrically controlled by imposing DC biases to the five electrodes on top of the system. Starting with an initial potential profile, the charge calculation is conducted in two ways. The electron density profile in the middle Si layer where electron spins are expected to reside is determined with electronic structure calculations based on the effective mass approximation, while the rest region that has almost no free carriers is handled with bulk physics to save computing power. Once the charge profile is determined, a 2D nonlinear Poisson solver is used to compute the new potential profile that will be used as an input for charge calculation in the next step. This self-consistent loop is conducted iteratively until the potential profile converges. In reality, the DQD device has a horseshoe-shaped local micromagnet to generate the laterally inhomogeneous magnetic field (*B*_*Z*_).^41^ As it is hard to directly include the micromagnet in the simulation domain, we take the spatial distribution of *B*_*Z*_ computed by Neumann and Schreiber^42^ and modify diagonal elements of the effective mass Hamiltonian. We note that more detailed description for device simulations can be found from one of our previous works.^35^ In reality, the electrostatic potential energy and corresponding spin confinement in Si QDs can be non-negligibly affected by size mismatches that happen during lithographical processes or atomistic natures like random atom distributions in SiGe layers.^43,44^ But here we do not include such factors in modeling, assuming that the DQD system is perfectly symmetric and does not suffer from undesirable atomistic effects.

Fig. 1(b) shows the charge stability diagram that is calculated when the barrier gate bias (*V*_B_) = 200 mV and the middle gate bias (*V*_M_) = 400 mV. When the left (*V*_L_) and right gate (*V*_R_) bias are low, the spin states in the middle layer are not occupied, so, for example, both QDs are empty when (*V*_L_, *V*_R_) = (525 mV, 555 mV) (the bias point A in the diagram). As we increase *V*_L_ and *V*_R_, however, the spin states shift lower in energy and, eventually, the lowest down-spin (|↓〉) state of each QD will be occupied by an electron. The right subfigures of Fig. 1(b) show the potential energy distributions in the middle Si layer at two bias points in the diagram. At the bias point A where both QDs are empty, the potential profile has two clear valleys where electrons can be confined. At the bias point *B* where *V*_L_ and *V*_R_ reach 540 and 570 mV, respectively, both |↓〉 states are filled and potential valleys do not exist any more due to electron screening. At this point, the DQD system is initialized to a 2-qubit |↓↓〉 state. In particular, we note the bias point *B* satisfies the symmetric biasing condition (*i.e.* the lateral potential profile in the middle Si layer becomes symmetric) that is known to be helpful to make spin operations robust to charge noise.^39^

### 2.2 DQD sensing protocol

The sensing protocol we would like to model with a Si DQD platform is based on the Ramsey interferometry measurement.^18,45,46^ For a 2-qubit system, the process for sensing externally given static magnetic field can be started by generating a superposition state |*ψ*_S_〉 with the initial state |↓↓〉 as shown in eqn (1).1
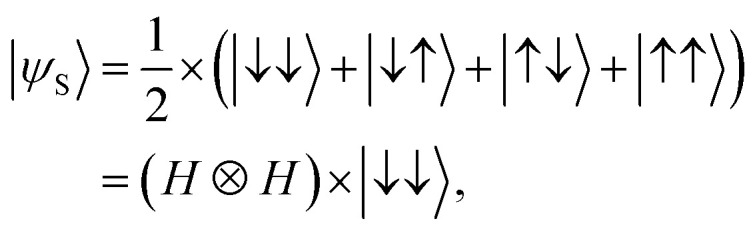
where *H* and ⊗ are a single-qubit Hadamard logic and a tensor product operation, respectively. Once the sensing source |*ψ*_S_〉 is prepared, we let |*ψ*_S_〉 evolve during time *T* in a native DQD system where the spin in each QD can be controlled independently (*i.e.* the exchange energy between two spins is zero). That is,

2 |*ψ*_T_〉 = exp(−*iH*_DQD_*T*) × |*ψ*_S_〉,

where *H*_DQD_ is the quantum Heisenberg model Hamiltonian for a 2-spin chain system whose elements are calculated with results of device simulations. Now, we conduct the post-operation against |*ψ*_T_〉 to get the measurable state |*ψ*_M_〉 as shown in eqn (3).

3 |*ψ*_M_〉 = (*I* ⊗ *H*) × CNOT × |*ψ*_T_〉,

where *I* is a single-qubit identity operation ((*H*, *I*) are conducted to the (control, target) qubit, respectively). *H*_DQD_ in eqn (2) is given by4
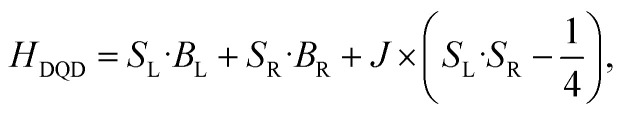
where (*S*_L_, *S*_R_) are the electron spin in the left & right QD, *J* is the exchange interaction between spins, and (*B*_L_, *B*_R_) are magnetic fields that two electron spins see. If (*B*_L_, *B*_R_) are static and oriented along the *Z*-direction, *H*_DQD_ in eqn (4) can be expressed as a 4 × 4 matrix with a set of {|↓↓〉, |↓↑〉, |↑↓〉, |↑↑〉} basis,5
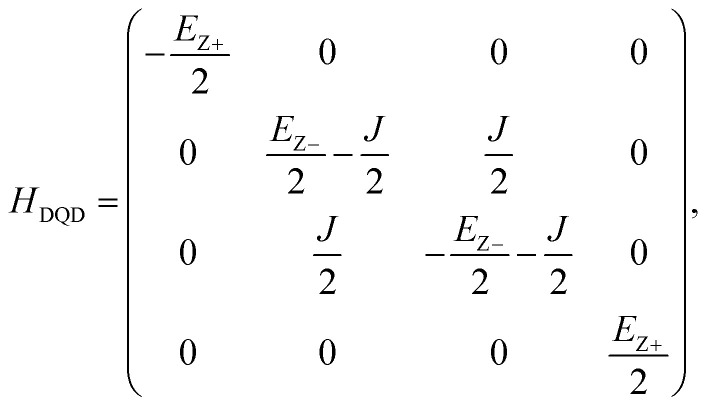
where *E*_Z+_ = *E*_ZR_ + *E*_ZL_ and *E*_Z−_ = *E*_ZR_ − *E*_ZL_ (*E*_ZR_ and *E*_ZL_ are the Zeeman-splitting energy of the spin in the right and the left QD of the DQD system, respectively). If two electron spins are perfectly isolated (*i.e. J* = 0), *H*_DQD_ in eqn (5) becomes a diagonal matrix, and the 2-qubit states |*ψ*_T_〉 and |*ψ*_M_〉 in eqn (2) and (3) can be rewritten as eqn (6) and (7),6
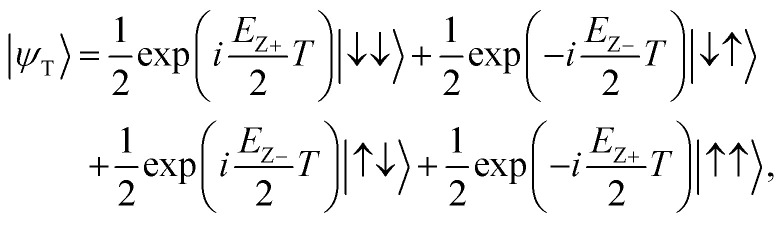
7
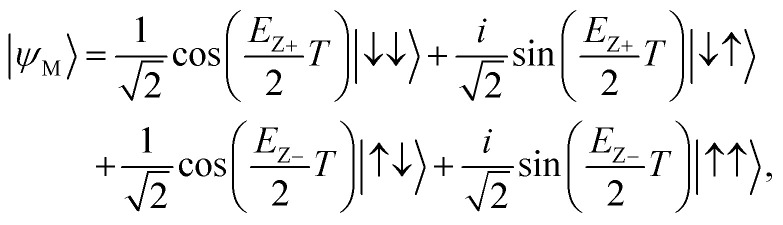
and we compute *E*_Z+_ and *E*_Z−_ by getting the state probabilities of |↓↓〉 and |↑↑〉 as a function of *T*, from which the quantity of *B*_L_ and *B*_R_ can be extracted.

## 3 Results and discussion

### 3.1 Conduction of pre- & post-operation

**Fig. 2:**
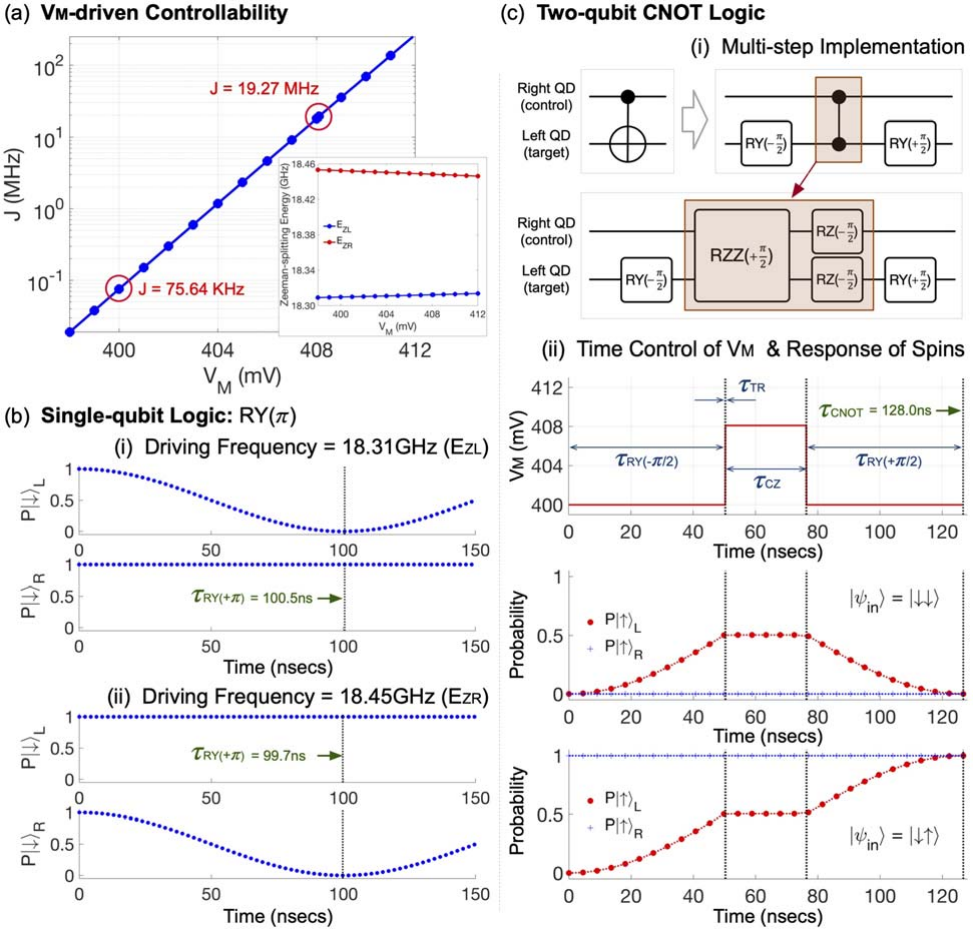
(a) The sensitivity of J to *V*_M_ that is simulated at (*V*_L_, *V*_R_, *V*_B_) = (540 mV, 570 mV, 200 mV). Here we choose two basing points (*V*_M_ = 400 mV and 408.1 mV where *J* = 75.64 kHz and 19.27 MHz, respectively) such that the first one can be used for individual qubit addressing while the other is for implementation of entangling logics. (b) Time-responses of spins in the DQD system that are calculated with *V*_M_ = 400 mV and a small time-varying magnetic pulse *B*_*Y*_(*t*) whose driving frequency is set to the Zeeman-splitting energy of the electron spin in the left (top) and the right QD (bottom). In this condition, spins rotate along the *Y*-direction, completing the Pauli-Y (RY(π)) logic in 100.5 ns and 99.7 ns for the left and the right QD, respectively. (c) (i) The multi-step implementation of a CNOT gate based on a sequential conduction of RY(−π/2) → CZ → RY(+π/2), and (ii) the real-time control of *V*_M_ needed for the multi-step CNOT operations and corresponding time-responses of QD spins. The RY logic can be implemented with *V*_M_ = 400 mV where spins weakly interact. The CZ logic needs a strong interaction mode (*V*_M_ = 408.1 mV), and can be implemented with no time-varying pulses.

In the Section 2.1 and Fig. 1(b), we described how the DQD system can be initialized to a |↓↓〉 state. The next step for designs of a sensing protocol is to define control signals that are necessary to conduct elementary gate operations to generate |*ψ*_S_〉 from the initial state (pre-operation), and |*ψ*_M_〉 from |*ψ*_T_〉 (post-operation). As shown in eqn (1) and (3), the pre- and post-operation require a single-qubit *H* and a 2-qubit CNOT gate. In the DQD system, any single-qubit logics must be driven in the regime where two electron spins weakly interacts so the spin in one QD can be manipulated independently of the spin in the other QD, while entangling gates generally require the regime where the interaction is strong. To secure biasing points that place the DQD system in these regimes, we first examine how the exchange interaction energy *J* between two QD spins is controlled with the bias *V*_M_ of the DQD system. Fig. 2(a) shows *J* as a function of *V*_M_ that is simulated at (*V*_L_, *V*_R_, *V*_B_) = (540 mV, 570 mV, 200 mV). In general, the spin interaction becomes stronger with a higher value of *V*_M_ as it lowers the potential barrier between two QDs. In particular, here we choose the point of *V*_M_ = 400 mV as a weak interaction mode where *J* remains in the order of kHz (75.64 kHz), and the point of *V*_M_ = 408.1 mV as a strong interaction mode where *J* reaches 19.27 MHz.

The *H* logic, which requires the DQD system to be in a weak interaction mode at *V*_M_ = 400 mV, can be completed with a sequential conduction of a RZ(+π) and a RY(+π/2) operation where RY and RZ indicate the rotation of a single electron spin around the *Y*- and the *Z*-axis, respectively. Since the RZ logic can be conducted instantly with software in reality,^11,41^ here we only need to discuss how the RY logic can be conducted to each spin in the DQD system. To rotate a single electron spin around the *Y*-axis, we need an additional time-varying magnetic pulse that is oriented along the *Y*-direction (*B*_*Y*_(*t*)), and the magnitude of *B*_*Y*_(*t*) should be small enough not to affect the electrostatic properties of the DQD system that are determined self-consistently with device simulations. After incorporating *B*_*Y*_(*t*) = *B*_o_ × cos(*ω*_D_*t*) into *H*_DQD_ in eqn (4), where *B*_o_ of 5.0 MHz is much smaller than the Zeeman-splitting energy of each QD spin that is determined by *B*_*Z*_ from the local magnet (Fig. 1(a)), we solve a time-dependent Schrödinger equation with an initial state |↓↓〉, and show results in Fig. 2(b). The time-response in the upper subfigure, which is obtained with *ω*_D_ equal to the Zeeman-splitting energy of the spin in the left QD (18.31 GHz), show that only the spin in the left QD oscillates, completing the Pauli-Y logic (RY(+π)) in ∼100.5 ns. When *ω*_D_ is set to the Zeeman-splitting energy of the spin in the right QD (18.45 GHz), only the spin in the right QD oscillates and the RY operation is a bit faster than the case of the left QD as the lower subfigure of Fig. 2(b) shows. For both QDs, the secured Pauli-Y logic shows a 99.98% fidelity in a noise-free condition.

The post-operation that is necessary to get the measurable state |*ψ*_M_〉 must involve a CNOT logic. While Zajac *et al.* demonstrated its successful implementation with a single time-varying magnetic pulse in the Si DQD system,^41^ one of our previous works shows the implementation based on a sequential conduction of RY and CZ gates makes the CNOT logic more robust to charge noise with little sacrifice in gating speed.^36^ This multi-step implementation of a CNOT logic is described in Fig. 2(c(i)), for which we need to apply *B*_*Y*_(*t*) for RY(±π/2) gating. The CZ gate that serves as an entangling step requires a strong interaction mode but can be achieved with no time-varying fields since the term exp(−*iH*_DQD_*T*) in eqn (2) with *H*_DQD_ in eqn (4) becomes a Ising ZZ (RZZ) coupling logic in the adiabatic regime.^36,47^ The real-time control of *V*_M_ for this multi-step CNOT gating and corresponding time-response of spins are shown in Fig. 2(c(ii)) where we assume switching *V*_M_ takes 0.1 ns. Results indicate the overall CNOT operation can be completed in ∼128.0 ns including two switching times (*τ*_TR_) with a noise-free fidelity of 99.07%. For RY with positive angles, we use *B*_*Y*_(*t*) as it is given in the previous paragraph. For negative angles, however, we need to introduce a phase shift to *B*_*Y*_(*t*) and *B*_*Y*_(*t*) = *B*_o_ × cos(*ω*_D_*t* + π).

### 3.2 Detection of unknown magnetic field

**Fig. 3:**
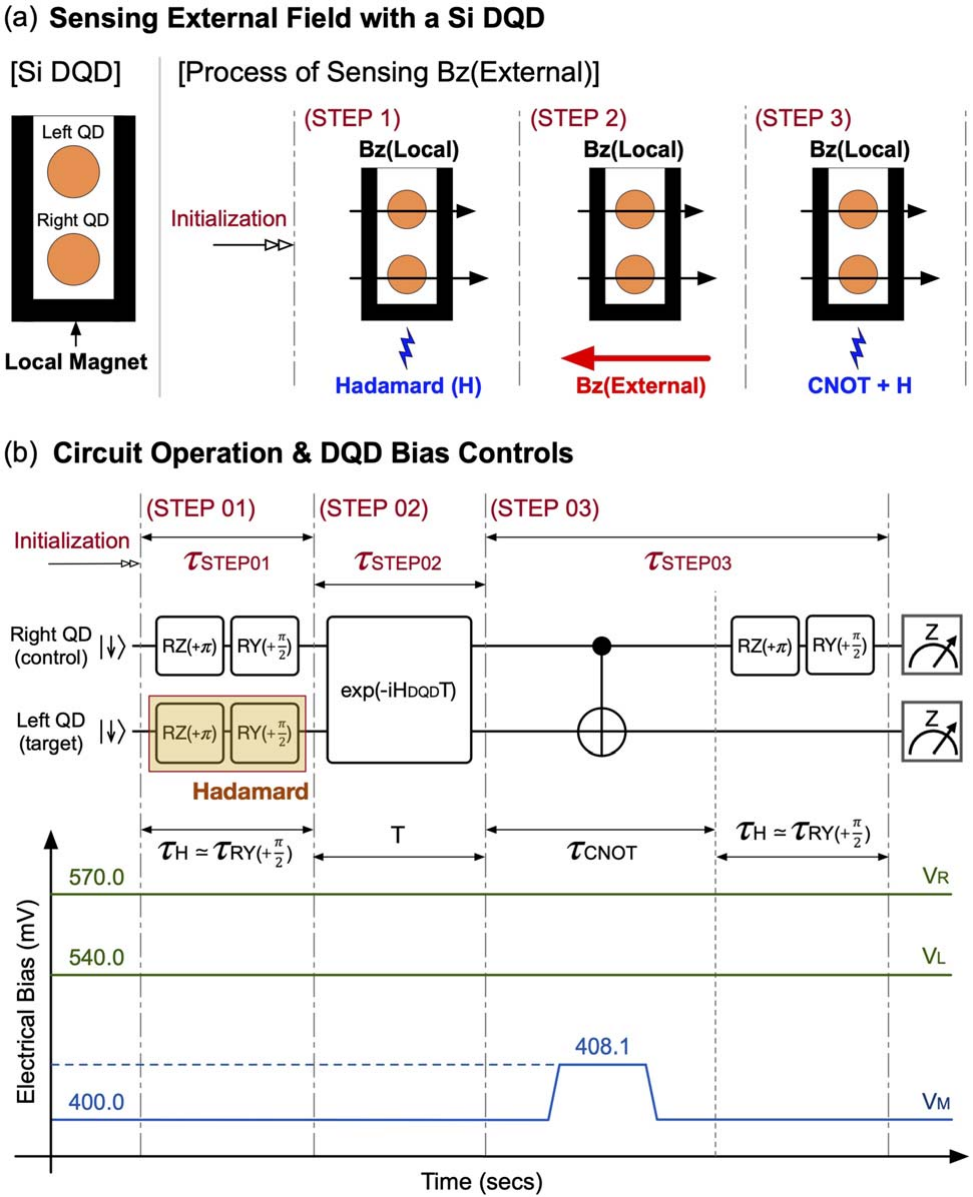
(a) The DQD-based sensing protocol involves the following steps: (i) after initialization of the DQD system, the pre-operation is conducted and |*ψ*_S_〉 is secured. Here, the sensing target (*B*_*Z*_(External)) is not given yet, and the operation is conducted under the field generated from a local magnet (*B*_*Z*_(Local)) of the DQD system. (ii) *B*_*Z*_(External) is given, and |*ψ*_S_〉 evolves to |*ψ*_T_〉 under a static field of *B*_*Z*_(External) + *B*_*Z*_(Local) in a weak interaction mode. (iii) The post-operation is done under *B*_*Z*_(Local) to get |*ψ*_M_〉, where the CNOT gating is implemented with the multi-step control shown in Fig. 2(c(i)). (b) Description of the sensing process in a circuit-level and the real-time control of electrical biases that is required to complete the process with the DQD system.

Since *H* & CNOT gating can be secured as described in the Section 3.1, the pre- & post-operation shown in eqn (1) and (3) can be done, and the focus of discussion now becomes the operational feasibility of a sensing protocol. For this purpose, we first present a conceptual scheme of the sensing process in Fig. 3(a), which will be simulated to examine how the detection of unknown external magnetic fields can be done with the Si DQD system. Here, the full process to be modeled consists of the three steps: (i) the pre-operation step is conducted in a weak-interaction mode under the magnetic field *B*_*Z*_ that stems from the local magnet (we denote this local magnetic field as *B*_*Z*_(Local)). (ii) Once the sensing source |*ψ*_S_〉 is obtained from the step (i), we let it evolve during the time *T* in the DQD system, which still needs to be in a weak-interaction mode to secure |*ψ*_T_〉. The external magnetic field (the sensing target, *B*_*Z*_(External)), being static and oriented along the *Z*-direction, is presented during this period, and the state evolves under the static field of *B*_*Z*_(Local) + *B*_*Z*_(External). (iii) The post-operation is conducted against |*ψ*_T_〉 under *B*_*Z*_(Local) and we get |*ψ*_M_〉. In Fig. 3(b), we describe a circuit-level description of the sensing process and the real-time control of biases that is required to complete the entire process (*V*_B_ = 200 mV & the transition time of *V*_M_ = 0.1 ns). Simulation results based on this bias control reveal the pre-operation time (*τ*_STEP01_) becomes ∼50.0 ns that is almost determined by the time needed to complete the RY(+π/2) logic against the spin in the left QD. The post-operation, which involves the multi-step CNOT logic, requires ∼178.0 ns (*τ*_STEP03_), so, in the modeling perspective, we recognize the sensing process can be finished in ∼(232 + *T*) nsec (considering the time required to complete the step (ii) (*τ*_STEP02_ = *T*)), and the operation would not be quite limited by the spin coherence time in DQD systems based on well purified Si wafers if *τ*_STEP02_ is not too large.

**Fig. 4:**
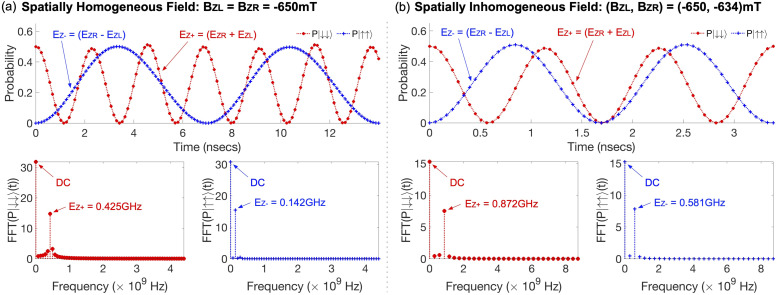
(a) The probability of |↓↓〉 and |↑↑〉 of the measurable state |*ψ*_M_〉 (*P*|↓↓〉 and *P*|↑↑〉) that are obtained as a function of *τ*_STEP02_ with the spatially homogeneous sensing target *B*_*Z*_(External) = −650 mT. Results of the Fast Fourier Transform (FFT) show the dominant frequency of *P*|↓↓〉 and *P*|↑↑〉 that are very close to the theoretical values of *E*_Z+_ and *E*_Z−_, supporting the solid feasibility of the DQD system as a sensing protocol for homogeneous fields (b) *P*|↓↓〉 and *P*|↑↑〉 simulated with the spatially inhomogeneous *B*_*Z*_(External) that marks −650 mT and −634 mT at the spot of the left QD (*B*_*Z*L_) and the right QD (*B*_*Z*R_), respectively. FFT-driven dominant frequencies of *P*|↑↑〉 and *P*|↓↓〉 also supports the solid operation of the sensing protocol for inhomogeneous fields. Note that all the FFT results given in this figure show a zero-frequency component, because *P*|↓↓〉 and *P*|↑↑〉 always have a constant term.

With the secured controls for the pre- & post-operation, now we can simulate the measurable state |*ψ*_M_〉 to get its state probability of |↓↓〉 and |↑↑〉 (*P*|↓↓〉 and *P*|↑↑〉) as a function of *τ*_STEP02_, during which |*ψ*_S_〉 evolves to |*ψ*_T_〉 under the sensing target. To examine the functionality of the DQD sensing protocol for spatially homogeneous fields, we simulate |*ψ*_M_〉 with a constant *B*_*Z*_(External) of −650 mT where the negative sign indicates *B*_*Z*_(External) is aligned in reverse of the direction of *B*_*Z*_(Local), and present the computational results in Fig. 4(a). As discussed in the Section 3.1 with Fig. 2(b), (*E*_ZL_, *E*_ZR_) = (18.31 GHz, 18.45 GHz) with *B*_*Z*_(Local), so the fields seen by the left (*B*_*Z*L_) and the right QD (*B*_*Z*R_) become +655.30 mT and +660.41 mT, respectively. With a *B*_*Z*_(External) of −650 mT, the new (*B*_*Z*L_, *B*_*Z*R_) should be (5.30 mT, 10.41 mT) and corresponding (*E*_ZL_, *E*_ZR_) = (148.32 MHz, 291.28 MHz) in a theoretical perspective. From eqn (7), we know that the oscillation frequencies of *P*|↓↓〉 and *P*|↑↑〉 are *E*_Z+_ and *E*_Z−_, respectively, and, by taking the Fast Fourier Transform (FFT) against simulated *P*|↓↓〉 and *P*|↑↑〉, we obtain the dominant frequency at 0.425 GHz for *P*|↓↓〉 and 0.142 GHz for *P*|↑↑〉, whose deviations with respect to their theoretical values are just ∼3.3% for *E*_Z+_ (=439.60 MHz) and ∼0.7% for *E*_Z−_ (=142.96 MHz).

Another case that we would like to computationally examine is the feasibility of the sensing protocol for spatially inhomogeneous fields. For this purpose, we assume that *B*_*Z*L_ and *B*_*Z*R_ driven with *B*_*Z*_(External) are different, and present the results simulated with a sensing target of (*B*_*Z*L_, *B*_*Z*R_) = (−650 mV, −634 mV) in Fig. 4(b). In this case, the new theoretical values of *E*_ZL_ and *E*_ZR_ with *B*_*Z*_(External) become 148.32 MHz and 739.16 MHz, respectively, since the state evolution in the step (ii) of the sensing process is done under (*B*_*Z*L_, *B*_*Z*R_) = (5.30 mV, 26.41 mV). FFT-driven dominant frequencies of the simulated state probabilities turn out to be 0.872 GHz and 0.581 GHz for *P*|↓↓〉 and *P*|↑↑〉, respectively, deviating by ∼1.7% (*P*|↓↓〉) and ∼1.6% (*P*|↑↑〉) from their theoretical values. We note that all the FFT results shown in Fig. 4 have a DC term since, from eqn (7), *P*|↓↓〉 and *P*|↑↑〉 of |*ψ*_M_〉 can be written as8
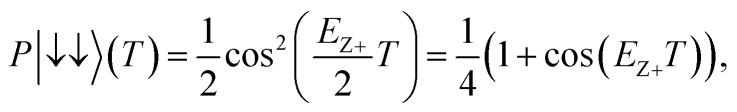
and9
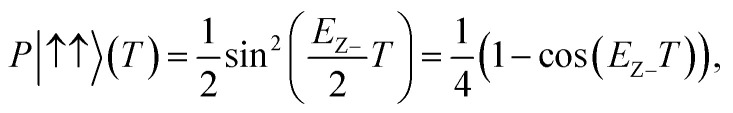
so both of them have a constant term of 1/4.

**Fig. 5:**
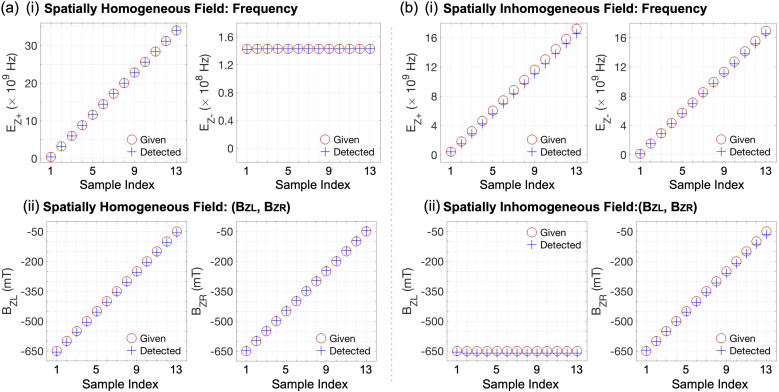
(a) Performance of the DQD sensing protocol when the sensing target is constant (spatially homogeneous). Being secured with *B*_*Z*_(External) (=*B*_*Z*L_ = *B*_*Z*R_) that varies from −650 mT to −50 mT with an incremental step of 50 mT, simulation results well confirm the operational feasibility of the sensing protocol, so the inaccuracies of *E*_Z+_'s & *E*_Z−_'s derived from simulated |*ψ*_M_〉 are just 0.49 ± 0.83% & 0.090 ± 0.21% of their theoretical values, and, accordingly, the detected *B*_*Z*L_'s & *B*_*Z*R_'s deviate by −5.72 ± 0.47 mT and 4.49 ± 0.46 mT from their given quantities, respectively. (b) Computational results driven with spatially inhomogeneous *B*_*Z*_(External) that is considered with *B*_*Z*L_ = −650 mV and *B*_*Z*R_ varying from −650 mT to −50 mT with a step of 50 mV. As similarly to the case of homogeneous targets, detected values here solidly follow given quantities. While the accuracies generally become worse than the case of homogeneous targets (*E*_Z+_'s: 6.70 ± 3.81%, *E*_Z−_'s: 2.20 ± 0.41%, *B*_*Z*L_'s: −10.02 ± 1.35 mT, and *B*_*Z*R_'s: −7.00 ± 5.27 mT), simulation results are fair enough to claim the operational feasibility of the protocol under inhomogeneous sensing targets.

Once we secure the values of *E*_Z+_ and *E*_Z−_ from *P*|↓↓〉 and *P*|↑↑〉 of |*ψ*_M_〉, the quantities of *B*_*Z*L_ and *B*_*Z*R_ introduced by *B*_*Z*_(External) can be easily determined since we know the quantities of *B*_*Z*L_ and *B*_*Z*R_ driven with *B*_*Z*_(Local) that is generated from the micromagnet). To investigate the performance of the DQD-based sensing protocol in a more general manner than what is shown in Fig. 4, we conduct simulations with more diverse conditions, and, for spatially homogeneous sensing targets, we consider *B*_*Z*_(External) (= *B*_*Z*L_ = *B*_*Z*R_) ranging from −650 mT to −50 mT with an incremental step of 50 mT, presenting corresponding computational results in Fig. 5(a). Here in general, the accuracy of *E*_Z+_ & *E*_Z−_ values determined from simulated |*ψ*_M_〉 is fairly nice, and the deviation from their theoretical values becomes just 0.49 ± 0.83% for *E*_Z+_'s and 0.090 ± 0.21% for *E*_Z−_'s. Simulation results also indicate that the detected *B*_*Z*L_'s and *B*_*Z*R_'s represent their given quantities quite well, revealing their associated inaccuracies mark −5.72 ± 0.47 mT and 4.49 ± 0.46 mT for *B*_*Z*L_'s and *B*_*Z*R_'s, respectively. Performance of the sensing protocol is also examined with spatially inhomogeneous sensing targets where *B*_*Z*R_ is again varied from −650 mT to −50 mV with a step of 50 mV. Computational results in Fig. 5(b) show that simulation-driven *E*_Z+_'s and *E*_Z−_'s still solidly follow their theoretical values when *B*_*Z*_(External) is spatially inhomogeneous, though they generally become less accurate than the cases of homogeneous targets (6.70 ± 3.81% and 2.20 ± 0.41% for *E*_Z+_'s and *E*_Z−_'s, respectively). Similarly, detected *B*_*Z*L_'s and *B*_*Z*R_'s represent given values well, but their inaccuracies turn out to be −10.02 ± 1.35 mT for *B*_*Z*L_ and −7.00 ± 5.27 mT for *B*_*Z*R_, being worse than the results obtained with homogeneous sensing targets.

Although the performance of sensing operations shows a non-negligible dependency on the sensing target, we claim the general pattern of detected fields shown in Fig. 4 and 5 is solid enough to support the feasibility of the Si DQD platform as a sensing protocol for magnetic fields, particularly including spatially inhomogeneous ones that are in principle impossible to be detected with a single QD device. All the computational results driven with simulations so far, however, are noise-free so, in the Section 3.3, we discuss how the sensing performance is affected when the Si DQD system is exposed to noisy conditions.

### 3.3 Sensitivity of sensing operation to charge noise

In reality, any quantum logic operations in Si QD systems must be affected by noise. The reason why highly purified Si^28^ wafers are required for designs of solid QPUs is also noise since nuclear spins in natural Si^29^ serve as a noise source, affecting the coherency of electron spins where qubits are encoded. Though it costs substantial money, however, this spin noise can be hugely suppressed by pushing the concentration of Si^29^ atoms in Si wafers below 50 ppm (0.005%),^1^ and nowadays highly purified 300 mm Si^28^ wafers are also available.^48^ Another type of noise inherent to Si is unintentionally fluctuating electric field, which is known as charge noise since electric field and charge distribution influence each other. Though there is the known rule of thumb for suppression of the spin noise in Si, no absolute ways to suppress Si charge noise are known so far up to our knowledge. There are studies reporting that the sensitivity of spin qubits to charge noise in Si can be reduced with fast measurements^38^ or controls of electrical biases,^36,39^ but they are not the ways to directly suppress charge noise. Accordingly, it becomes crucial to explore how the sensing protocol behaves under charge noise of substantial magnitudes.

**Fig. 6:**
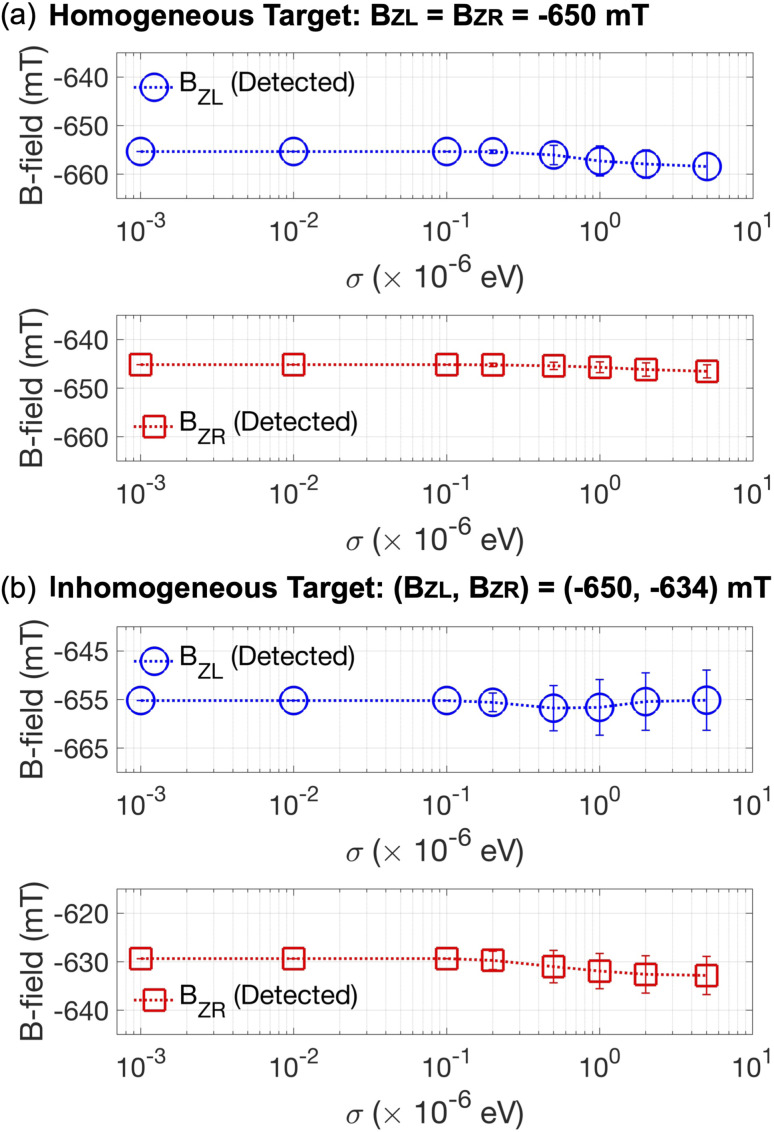
(a) The sensitivity of sensing operations to charge noise simulated with a constant sensing target of −650 mT. Even in the worst case where the noise strength (*σ*) reaches 5.0 μeV, the average quantities of detected *B*_*Z*L_'s and *B*_*Z*R_'s differ by just −3.07 mT and −1.37 mT from their noise-free values, respectively. (b) The operational sensitivity examined for an inhomogeneous target (*B*_*Z*L_ = −650 mT, *B*_*Z*R_ = −634 mT). The stability of sensing operations here is also well supported since the average difference between noisy and noise-free quantities at *σ* = 5.0 μeV become 0.08 mT for *B*_*Z*L_ and −3.48 mT for *B*_*Z*R_.

To computationally investigate sensing operations under noisy conditions, we conduct the same set of simulations as those employed to drive the results in Fig. 4, but here we effectively consider the effect of charge noise on the electronic structure of the Si DQD system by introducing random fluctuation to the potential energy distribution that is self-consistently determined with device simulations. The potential fluctuation is generated in every grid of the 2D simulation domain with a zero-mean Gaussian distribution. The standard deviation (*σ*) of this random potential energy, which indicates the strength of charge noise, is chosen in the range from 10^−3^ μeV to 5.0 μeV, where the maximal value of *σ* well reflects the strength of charge noise reported for physical Si devices.^38^ For each value of considered *σ*'s, a total of 200 simulations are conducted to incorporate the random nature of noisy potential energy into sensing operations. In Fig. 6(a), we show the results obtained when a constant field of *B*_*Z*L_ = *B*_*Z*R_ = −650 mT is given as a sensing target. The noise-free values of detected *B*_*Z*L_ and *B*_*Z*R_ are −655.34 mT and −645.17 mT, respectively, and the effect of charge noise on these values is not quite remarkable when *σ* ≤ 0.1 μeV. Stronger noise, however, obviously affects the sensing operation, and, in the worst case when *σ* = 5.0 μeV, detected *B*_*Z*L_ and *B*_*Z*R_ become −658.41 ± 2.71 mT and −646.54 ± 1.34 mT, respectively. Simulation results given in Fig. 6(b), which are obtained with an inhomogeneous target of (*B*_*Z*L_, *B*_*Z*R_) = (−650 mV, −634 mV), also indicate the noise-free detected quantities (*B*_*Z*L_ = −655.22 mT, *B*_*Z*R_ = −629.35 mT) are not quite affected by charge noise when *σ* ≤ 0.1 μeV, but *B*_*Z*L_ and *B*_*Z*R_ reach −655.14 ± 6.20 mT and −632.83 ± 3.95 mT, respectively, when *σ* = 5.0 μeV.

Although the results shown in Fig. 6 clearly indicate the absolute truth that a sensing protocol device designed with the Si DQD system cannot be free from charge noise, the sensitivity of sensing operations is not quite strong in general so the averages of detected *B*_*Z*L_'s and *B*_*Z*R_'s with *σ* = 5.0 μeV differ by just 0.47% (−3.07 mT) and 0.21% (−1.37 mT) in magnitude from their noise-free values under a homogeneous target. The noise-sensitivity of operations does not change much with an inhomogeneous sensing target, such that the average difference between noisy and noise-free results at *σ* = 5.0 μeV becomes 0.01% (0.08 mT) for *B*_*Z*L_ and 0.55% (−3.48 mT) for *B*_*Z*R_, supporting the operational stability of the DQD-based sensing protocol.

## 4 Conclusions

In this work, we computationally examined the operational feasibility of a sensing protocol for static magnetic fields that is designed with electron spins in the electrically controlled silicon (Si) quantum dot (QD) platform. With an empirical description of electronic structures augmented by bulk physics, we rigorously simulate a realistically sized double QD (DQD) device that can have up to two electron spin quantum bits (qubits), securing control signals required for initialization of the DQD system and implementation of single- & two-qubit gates that are essential for sensing operations. End-to-end operations of the protocol whose logic is based on the simple Ramsey interferometry measurement, are extensively tested against spatially homogeneous and inhomogeneous fields. Although the performance becomes generally better for detection of homogenous fields, the overall results are fairly good enough to support the feasibility of the Si QD platform as a sensing device even when the process is under substantial charge noise. The protocol studied in this work uses a superposition state as a sensing source (a Type-II quantum sensor according to the classification scheme reported by Degen *et al.*^18^), so its superiority in performance to classical sensors is somewhat ambiguous unlike the case where entangled states act as sensing sources. Another point that is not studied with modeling is the measurement process that would be a source of errors in reality. Nevertheless, the design & engineering details that this work presents can contribute to expanding the application scope of electron spins in Si whose main application so far is quantum computation.

## Data availability

The authors declare that the data supporting the findings of this study, which are mainly the computational results obtained with an in-house device simulation code, are available in the paper.

## Author contributions

HR conceived the project, conducted simulations, analyzed simulation results, and prepared for the first draft of the manuscript. JR conducted simulations and analyzed simulation results. All the authors revised the draft and prepared for the final manuscript.

## Conflicts of interest

The authors declare no conflicts of interest.
